# Research on Fabrication of Phononic Crystal Soft-Supported Graphene Resonator

**DOI:** 10.3390/nano14020130

**Published:** 2024-01-05

**Authors:** Xiande Zheng, Ying Liu, Jiapeng Zhen, Jing Qiu, Guanjun Liu

**Affiliations:** College of Intelligence Science and Technology, National University of Defense Technology, Changsha 400713, China; zxd@nudt.edu.cn (X.Z.); liuying@nudt.edu.cn (Y.L.); zhenjiapeng23@nudt.edu.cn (J.Z.); qiujing@nudt.edu.cn (J.Q.)

**Keywords:** graphene resonator, phononic crystal soft support, energy dissipation dilution, quality factor

## Abstract

In aviation, aerospace, and other fields, nanomechanical resonators could offer excellent sensing performance. Among these, graphene resonators, as a new sensitive unit, are expected to offer very high mass and force sensitivity due to their extremely thin thickness. However, at present, the quality factor of graphene resonators at room temperature is generally low, which limits the performance improvement and further application of graphene resonators. Enhancing the quality factor of graphene resonators has emerged as a pressing research concern. In a previous study, we have proposed a new mechanism to reduce the energy dissipation of graphene resonators by utilizing phononic crystal soft-supported structures. We verified its feasibility through theoretical analysis and simulations. This article focuses on the fabrication of a phononic crystal soft-supported graphene resonator. In order to address the issues of easy fracture, deformation, and low success rate in the fabrication of phononic crystal soft-supported graphene resonators, we have studied key processes for graphene suspension release and focused ion beam etching. Through parameter optimization, finally, we have obtained phononic crystal soft-supported graphene resonators with varying cycles and pore sizes. Finally, we designed an optical excitation and detection platform based on Fabry–Pérot interference principle and explored the impact of laser power and spot size on phononic crystal soft-supported graphene resonators.

## 1. Introduction

In recent years, there has been a pressing need for high-performance sensors in fields such as aviation and aerospace. Exploring the application of new materials to enhance sensor performance has become an effective approach. Graphene has attracted worldwide attention [1,2,3] since it was first prepared in 2004 by Andre Geim and Konstantin Novoselov of the University of Manchester using the micromechanical stripping method [4]. Graphene consists of a single atomic layer with ultra-thin characteristics, as well as excellent thermal and electrical conductivity. The Young’s modulus of graphene is up to 1 GPa. Currently, numerous studies focus on sensing applications utilizing graphene-sensitive materials. Among these, resonant sensors that employ graphene resonators as sensitive units are a significant research focus in the field of graphene sensing. Based on extensive domestic and international research, graphene resonators, as sensitive units, have very high sensitivity due to the extremely thin thickness of graphene. Graphene resonators offer several advantages, including a wide frequency response, rapid response speed, low power consumption, and excellent biocompatibility. These characteristics make them promising for applications in low-voltage measurement, molecular and atomic mass detection, and biosensing [5,6,7,8,9].

However, currently, the quality factor of graphene resonators at room temperature is generally low [10,11,12,13,14,15,16,17,18,19,20,21,22,23]. Therefore, enhancing the quality factor of graphene resonators has become an urgent research issue. The quality factor is directly proportional to the ratio of the total energy stored in the resonator to the energy dissipated per vibration cycle. Reducing energy dissipation is a crucial method for enhancing the quality factor of resonators. In recent years, the energy dissipation dilution theory has been effectively utilized to enhance the quality factor of silicon nitride resonators [24,25,26,27,28,29,30,31,32,33,34,35]. Whether this theory can be applied to graphene resonators to enhance their quality factor requires further research.

In our previous studies, to solve the problem of the low-quality factor of graphene resonators at room temperature, we have developed a dissipative dilution theoretical model for graphene resonators and preliminarily designed phononic crystal soft-supported structures. In this article, we deeply analyzed the dissipation distribution characteristics and revealed the main concentration of energy dissipation in graphene resonators. We then proposed a new mechanism for suppressing energy dissipation by using phononic crystal soft-supported structures. Based on the new mechanism and optimized structure, we designed the overall fabrication process flow of phononic crystal soft-supported graphene resonators. During the fabrication flow, when suspending the graphene from the support layer, graphene is easy to break, so we studied the key processes for graphene suspension release. Meanwhile, to address the issues of deformation and low success rate in the etching process of phononic crystal patterns, we explored key processes for focus ion beam etching of phononic crystal soft-supported graphene resonators. At last, an optical excitation and detection platform based on the Fabry–Pérot interference principle was designed. Based on the platform, how the laser power and spot size influence the central temperature and fundamental frequency of phononic crystal soft-supported graphene resonators has been studied through finite element simulation.

## 2. Methodology

Graphene possesses an extremely high Young’s modulus and lightweight properties at the atomic level. There has been a debate over whether modeling graphene resonators under high prestress conditions is suitable for thin plate or thin film models. When subjected to high stress, experimental research tends to support graphene as a viscoelastic material, and the bending stiffness cannot be ignored. Based on the classical thin plate theory [36,37,38] and the Zener anelastic model [39,40], a complex-valued Young’s modulus is introduced, resulting in a phase lag between the strain and stress: $\tilde{E}\equiv E_{1}+iE_{2}$. Then, for each oscillating cycle, the total energy $U_{mn}$ and dissipation energy $\Delta U_{mn}$ can be calculated. Finally, the quality factor $Q_{mn}$ can be expressed as:

(1) $$Q_{mn}=\frac{2\pi U_{mn}}{\Delta U_{mn}}=A_{mn}\times Q_{\mathrm{int}}$$

in which $A_{mn}$ is the quality factor amplification coefficient, and $Q_{\mathrm{int}}$ is the intrinsic material quality factor: $Q_{\mathrm{int}}=E_{1}/E_{2}$.

To obtain the analytical solution of Equation (1), for a specific mode (*m*, *n*), $A_{mn}$ can be divided into two parts: the distribution term $A_{mn}^{\mathrm{distributed}}$ is the region far from the fixed support boundary, and the fixed support term $A_{mn}^{\mathrm{clamping}}$ represents the fixed support boundary and its adjacent region. The overall quality factor amplification coefficient can be calculated as:

(2) $$A_{mn}={\left[{\left(A_{mn}^{\mathrm{distributed}}\right)}^{-1}+{\left(A_{mn}^{\mathrm{clamping}}\right)}^{-1}\right]}^{-1}={\left[\frac{\rho \omega^{2}D}{h\sigma^{2}}+\frac{hS_{\mathrm{cla}}\sigma_{\mathrm{cla}}^{2}}{\rho D\omega^{2}\iint_{\mathrm{cla}}W{\left(x,y\right)}^{2}dxdy}\right]}^{-1}$$

For a more detailed derivation of the formula, please refer to [41].

The energy dissipation at each position on a graphene resonator is typically assessed by using normalized curvature:

(3) $$C_{N}=\left|\partial^{2}W/\partial x^{2}+\partial^{2}W/\partial y^{2}\right|U_{mn}^{-1/2}$$

In fact, the square of the normalized curvature is inversely proportional to the quality factor amplification coefficient when integrated over the entire surface of the graphene resonator: $A_{mn}^{-1}=D/2\times \int \int C_{N}^{2}dxdy$. The higher the normalized curvature, the lower the quality factor amplification coefficient and the greater the energy dissipation. Combining finite element simulation, we investigated the normalized curvature distribution of boundary-fixed supported square graphene and silicon nitride resonators with a side length of 9.6 μm and an initial applied stress of 1 GPa. Table 1 presents the specific parameters of graphene and silicon nitride utilized in the simulation.

**Figure 1:**
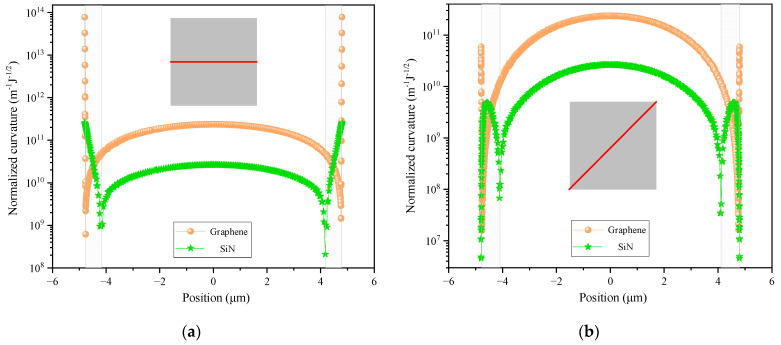
Normalized curvature distribution of the first mode of square graphene and silicon nitride resonators (**a**) along the centerline of the x-axis and (**b**) along the diagonal. The red lines indicate the horizontal centerline and the diagonal respectively.

To compare the normalized curvature inside and on the edges of the resonator, and to compare the differences between the two materials of resonators, the normalized curvature distributions of the first-order mode along the horizontal centerline and diagonal were depicted in Figure 1a,b, respectively. The orange sphere represents the normalized curvature distribution of the graphene resonator, while the green pentagram represents the normalized curvature distribution of the silicon nitride resonator. The schematic diagram shows the horizontal centerline and diagonal in the illustration. From Figure 1, it can be observed that the maximum normalized curvature on the edge of the first mode of the graphene resonator is three orders of magnitude larger than the normalized curvature at the inner center, whereas the difference in this aspect of the silicon nitride resonator is only one order of magnitude. At the same time, the slope of the normalized curvature transition from the edge to the interior of the silicon nitride resonator is much lower than that of the graphene resonator. This indicates that the normalized curvature of graphene is highly concentrated at the edge, while the normalized curvature of the silicon nitride resonator is concentrated at the edge and within a small range around it (side length ranging from 8.6 to 9.6 μm within the concentric square ring), as shown in the semi-transparent gray area in Figure 1a,b.

The energy dissipation of graphene resonators is highly concentrated at the fixed support boundary, indicating the need for measures to suppress the energy dissipation at the fixed support boundary. The energy dissipation at the fixed edge is caused by the phonon tunneling loss in the external loss mechanism, often known as anchoring or fixed loss. The fundamental physical mechanism is that, when an elastic body vibrates, its fixed end generates shear forces and bending moments, which serve as excitation sources to stimulate elastic waves on the base and propagate to infinity for dissipation. In 2008, Ignacio Wilson Rae [42,43] first proposed the concept of phonon tunneling loss and derived the expression for the reciprocal of the resonator quality factor under the influence of phonon tunneling loss:

(4) $$\frac{1}{Q}=\frac{\pi \int_{q}{\left|\int_{S}\;d\bar{S}\cdot \left(\sigma_{q}^{\left(0\right)}\cdot {\bar{u}}_{R}^{\prime }-\sigma_{R}^{\prime }\cdot {\bar{u}}_{q}^{\left(0\right)}\right)\right|}^{2}\times \delta \left[\omega_{R}-\omega (q)\right]}{2\rho_{s}\rho_{R}\omega_{R}^{3}}$$

Equation (4) indicates that for, the integral term, energy dissipation can be reduced by decreasing the contact area between the resonator and the supporting structure, as the integrated quantity is a square term greater than zero. Additionally, for the frequency difference correlation function term, phonon tunneling loss can be minimized by preventing frequency overlap between the resonator and the supporting structure.

In the past few decades, numerous theoretical and experimental studies have proposed various methods to mitigate this loss. These methods include reducing the contact area between resonators and support structures and avoiding frequency overlap between them. Although it can reduce phonon tunneling loss, there are still one or two issues: (1) The placement of the direct support structure significantly affects the loss. Inaccurate positioning cannot reduce phonon tunneling loss. (2) The indirectly supported suspension structure has its own vibration mode, which contaminates the original vibration spectrum and generates additional vibration modes. Relatively speaking, similar to photonic crystals [44], phononic crystal structures offer a means to directly define a frequency spectrum region without vibration, known as a phonon bandgap. In this bandgap, elastic waves cannot propagate, and any excitation within the bandgap frequency range will be exponentially suppressed [45,46], without causing the aforementioned two problems. This article proposes a new mechanism to reduce the energy dissipation of graphene resonators, using phononic crystal soft-supported structures to mitigate phonon tunneling losses, thereby improving the quality factor of graphene resonators.

## 3. Materials and Methods

### 3.1. Research on Fabrication Process Flow

**Figure 2:**
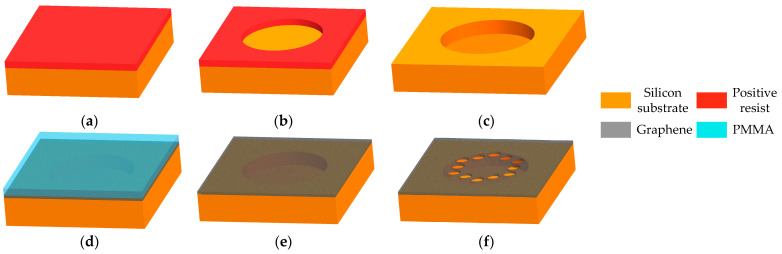
Fabrication process of phononic crystal soft-supported graphene resonator: (**a**) spin-coated photoresist; (**b**) UV lithography; (**c**) deep reactive plasma etching; (**d**) graphene transfer; (**e**) remove PMMA; (**f**) focused ion beam etching.

According to the new mechanism, we have designed the structure of phononic crystal soft-supported graphene resonator in triangular lattice with central defect and verified the feasibility of suppressing phonon tunneling losses through phononic crystal structures via finite element simulation, as illustrated in Figure 1 and Figure 2 in our previous work [41]. The core principle is utilizing phononic crystal soft-supported structure to localize the vibration energy within the central defect area, thereby preventing energy from dispersing to the boundary support area. Moreover, we have further optimized the structure of phononic crystals to achieve higher quality factors, as depicted in Figures 1, 4, 6 and 9 in Reference [41]. This article mainly focuses on how to fabricate the phononic crystal soft-supported graphene resonator based on the structure designed in [41], with the only difference being the fixed boundary in a circular shape. The final design of the fabrication process flow is shown in Figure 2.

We included the following steps:

(a): Clean silicon substrate (300 μm thickness) with ultrasonic cleaning machine, then spin-coating a layer of photoresist (AZ4620, AZ Electronic Materials, Tokyo, Japan) to prepare for UV exposure;
(b): Set appropriate parameters in the UV lithography machine (SUSS MA6, SUSS MicroTec, Schleissheimer, Germany), perform UV exposure, followed by development, fixation, and post-drying. Place the substrate under a metallographic microscope for observation, mainly observing whether the shape and size of the photolithography feature pattern are normal. If not, analyze whether the exposure dose, baking temperature, and development time need to be adjusted. Finally, transfer the pattern to be etched on the mask onto the photoresist;
(c): Considering the large aspect ratio up to 10:1, deep reaction plasma etching machine (SPTS Omega LPX Rapier, SPTS Technologies Ltd., Newport, UK) was used to etch the silicon substrate. After transferring the etched patterns on the positive photoresist onto the silicon substrate, remove residual photoresist; finally, obtain a perforated silicon substrate to prepare for achieving a graphene suspension cavity;
(d): The graphene selected in this article is one-step transfer graphene (Trivial Transfer Graphene, TTG) 9. Compared with traditional CVD copper-based graphene, this product eliminates the steps of spin-coating PMMA and etching copper, reduces the operating steps, reduces the number of graphene wrinkles, and avoids the problem of inorganic residue in the copper-etching step. Release TTG into deionized water, let it stand in water for two hours, then transfer it onto the perforated silicon substrate through wet transfer; at last, fully dry the graphene by gradually increasing the temperature;
(e): Remove polymethyl methacrylate (PMMA) from the surface of graphene through back float method and high-temperature annealing method, release graphene into suspension, and achieve the suspended cavity structure of graphene;
(f): Using the focused ion beam (FIB) etching machine (FEI Scios Dual Beam, Thermo Fisher Scientific, Waltham, MA, USA), phononic crystal structures were etched onto suspended graphene; ultimately, a phononic crystal soft supported graphene resonator was prepared.

The successful implementation of each step in the above process flow is crucial for the successful preparation of the final phononic crystal soft-supported graphene resonator sample. Micro–nano-processing is highly sensitive to changes in process parameters. Therefore, the specific parameters of each process step need to be verified and optimized repeatedly. In actual sample preparation, it is essential to maintain strict control over the preparation environment and adhere closely to the process flow and parameters for implementation.

**Figure 3:**
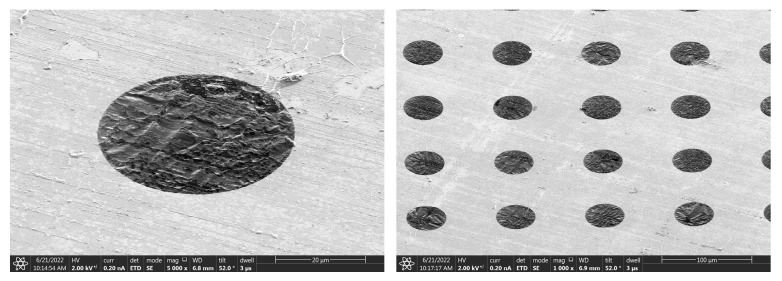
Observation of wrinkles on the surface of graphene under scanning electron microscopy.

The steps (a)–(d) in the above process flow represent the traditional process for preparing suspended graphene. It should be noted that graphene is susceptible to wrinkling and stacking during the transfer process, as depicted in Figure 3. The key influencing factor is whether the baking in step (d) is sufficient. In response to this issue, this article conducted experiments to control the temperature during the baking process to reduce the occurrence of wrinkles.

In actual operation, the specific step-by-step temperature control steps are as follows: bake at 50 °C, 60 °C, 70 °C, 80 °C, 90 °C, and 100 °C for 5 min each, and, finally, bake at 150 °C for 10 min. By employing a gradual and slow heating process to eliminate moisture from graphene and silicon wafers, and using PMMA to melt and flatten at high temperatures to further smooth out wrinkles on the graphene surface, a high-quality PMMA/graphene/silicon substrate composite structure is achieved. This prepares for the subsequent step of removing the PMMA support layer and releasing the graphene into the air.

### 3.2. Research on Suspended Release of Graphene

At step (e), to address the issues of easy breakage and low success rate of graphene suspension release, we studied the key processes for releasing graphene suspension. Based on the susceptibility of suspended graphene films to easy breakage in traditional impregnation methods, the back float method and high-temperature annealing method were chosen to enhance the success rate of graphene suspension release.

#### 3.2.1. Back Float Method

During the wet method process, which involves removing PMMA with a chemical reagent solution, the graphene film is highly susceptible to rupture. However, microscopic observation reveals that the graphene film rarely ruptures before the PMMA support layer is removed. The film rupture mainly occurs during the release process, when the liquid begins to dry from the central region, creating a new three-phase interface that is formed between the liquid, graphene film, and air. The film undergoes sudden additional stress and ruptures.

**Figure 4:**
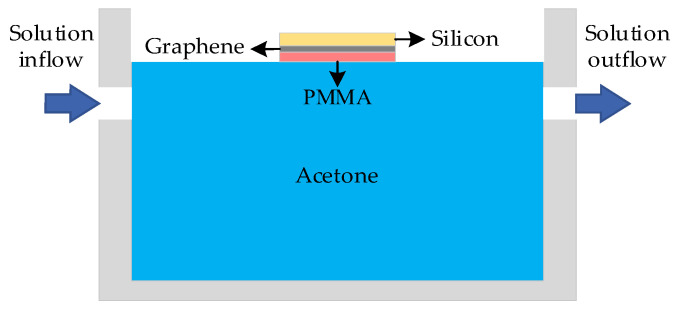
Schematic diagram of PMMA removal by back float method.

To address this issue, this article introduces the back float method, which involves inverting the sample so that the PMMA faces downwards and comes into contact with the etching solution. This method can prevent the etching solution from flowing into the hole, thus avoiding the formation of a three-phase interface. The schematic diagram of PMMA removal using the back float method is depicted in Figure 4.

**Figure 5:**
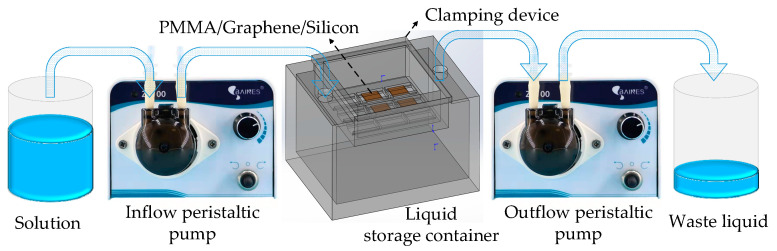
Composition diagram of the back float method device.

This article addresses the issue of controlling liquid level height by introducing two peristaltic pump modules to optimize the backflow process. One peristaltic pump controls the rate of solution inflow, while the other peristaltic pump controls the rate of solution outflow. The schematic diagram of the optimized device composition is depicted in Figure 5.

**Figure 6:**
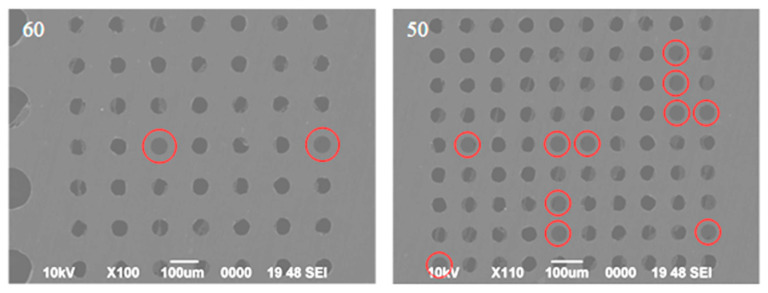
Partial results of graphene suspension release through back float method. All successfully suspended graphene is highlighted in red circle.

Figure 6 displays partial results of the release of graphene suspension observed by a scanning electron microscope (SEM) (JSM-6390, JEOL, Yokyo, Japan). For 6–8-layer graphene, several graphene sheets have been successfully suspended over circular holes with a diameter of 60 μm, and more than ten graphene sheets have been successfully suspended over circular holes with a diameter of 50 μm. All successfully suspended graphene is highlighted in red circle. As the diameter of the circular hole decreases, more successfully suspended graphene becomes accessible.

While the back float method has enhanced the success rate of suspending graphene to some extent, its ability to improve the success rate of large-diameter one is limited. Therefore, this article continues to explore the dry method for removing PMMA.

#### 3.2.2. High-Temperature Annealing Method

PMMA decomposes into gases and volatile substances at high temperatures. Therefore, high-temperature annealing can be utilized to eliminate PMMA residues and other contaminants from graphene. This method is known for its rapid and cost-effective characteristics. In a vacuum environment, the lack of gas molecules results in relatively fewer defects generated by vacuum annealing. This article selects a tubular annealing furnace (L4508, Semic Microelectronics Equipment Co., Ltd., Qingdao, China) capable of creating a vacuum environment for the dry removal of PMMA.

Three main parameters influence the success rate of suspending graphene during high-temperature annealing in a vacuum environment: heating rate, annealing temperature, and annealing time. Due to the thermal shrinkage and cold expansion characteristics of graphene, high temperatures induce stress in graphene. Excessive heating rates can result in a sharp increase in stress within graphene, leading to its rupture. Similarly, inadequate annealing temperature and duration can also lead to incomplete removal of PMMA. Through exploration, the optimized heating rate, annealing temperature, and annealing time are 12 °C/min, 500 °C, and 20 min, respectively.

**Figure 7:**
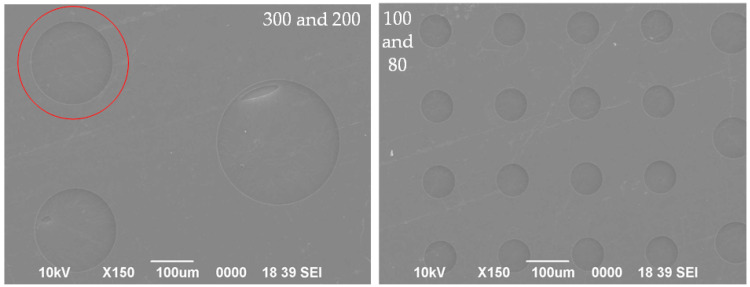
Partial results of graphene suspension release through high-temperature annealing. The red circle indicates a suspended graphene sample in 200 μm pore size without obvious defects.

Figure 7 displays partial results of releasing of graphene suspension. For 6–8-layer graphene, significant damage is still visible in graphene with a pore size of 300 μm, while a suspended graphene sample with a 200 μm pore size shows no obvious defects, as indicated by a red circle. At the same time, the suspension of graphene on the 100 μm and 80 μm pore sizes is relatively effective, especially on the 80 μm pore size, with a suspension success rate of nearly 100%.

After conducting statistical analysis on the suspension status of several graphene sheets mentioned above, the success rates of graphene suspension for each pore size (6–8-layer graphene) are presented in Table 2. Likewise, the smaller the pore size, the greater the success rate of suspension.

### 3.3. Research on Focused Ion Beam Etching

**Figure 8:**
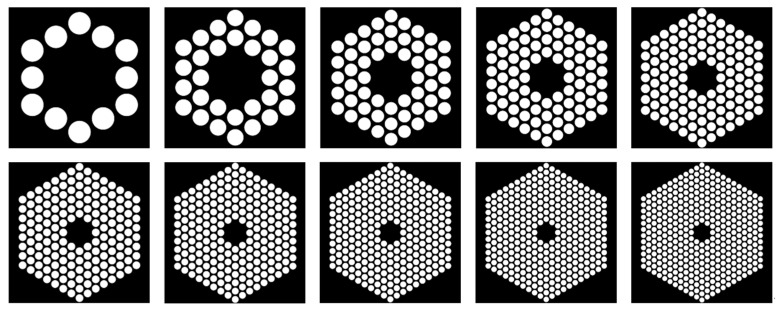
FIB etching patterns with phononic crystal structure.

In step (f), to address the issues of easy fracture, deformation, and low success rate in the etching process of phononic crystal patterns, we studied key processes for FIB etching of phononic crystal soft-supported graphene resonators. FIB etching technology is a microfabrication technique that utilizes an electrostatic lens to focus ion beams into very small sizes. The ion beam of the FIB system is extracted from a liquid gallium ion source. According to the research findings in [41], we have designed the FIB etching patterns with phononic crystal structure as depicted in Figure 8. In the ten patterns, the structures are composed of 1 to 10 cycles of phononic crystal cells, arranged from left to right and from top to bottom.

We investigated several factors that influence the success rate of FIB etching. Specifically, we focused on the impact of surface wrinkles, the number of graphene layers, the suspended release mode, and etching cycles to optimize etching parameters.

#### 3.3.1. Surface Wrinkles

**Figure 9:**
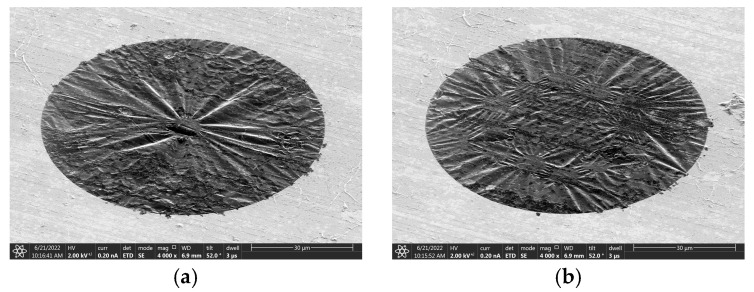
FIB etching results of (**a**) simple circular hole and (**b**) 1 cycle of phononic crystal cells.

Before etching the specific phononic crystal structure pattern, we conducted an etching experiment using a simple circular pattern. The etching result is shown in Figure 9a, revealing that the surface of the graphene is relatively loose. After bombarding the surface with FIB in a circular pattern, the center of the graphene collapses downward under the force of ion bombardment, causing numerous wrinkles to form around it. Cracks have appeared on the surface of the graphene before the complete circular pattern was etched.

After attempting to etch a simple circular pattern, we conducted subsequent etching experiments using the simplest 1-cycle phononic crystal pattern (as depicted in the first image in Figure 8). The etching result is shown in Figure 9b. It is evident that, after the FIB hits the surface of graphene, a downward collapsed phononic crystal structure pattern emerges, accompanied by numerous sinking wrinkles around it. Similarly, before the complete pattern is etched, graphene has already locally ruptured.

By tracing the transfer process of graphene, it was found that the wrinkling occurred because the graphene/PMMA composite structure was not fully dried after being lifted from water using silicon wafers in the initial stage, leading to inadequate flattening of PMMA. During the process of transferring graphene, it is essential to use a gradual heating method to ensure thorough post-baking of the graphene/PMMA composite structure. On one hand, it can prevent the fracture of suspended graphene, while, on the other hand, it can also reduce the surface wrinkles of graphene and improve the success rate of FIB etching.

#### 3.3.2. Number of Graphene Layers

Using the optimized drying method, we conducted FIB etching experiments on 6–8 layers of graphene. Optimizing the drying method effectively addresses the issue of surface relaxation. However, there are still challenges related to deformation and damage after the penetration of graphene, which hinders the attainment of a complete phononic crystal structure.

**Figure 10:**
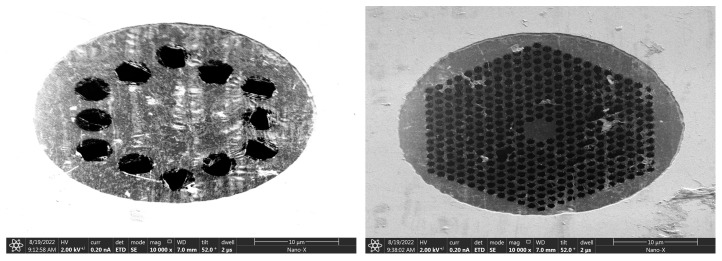
FIB etching results of 3–5 layers of graphene.

Further, we attempt to reduce the number of graphene layers from 6–8 to 3–5. The FIB etching results are shown in Figure 10. It can be observed that the phononic crystal patterns have been successfully etched into the 3–5 layers of graphene. Not only can a simple one-cycle phononic crystal structure pattern be etched, but even the most complex 10-cycle phononic crystal structure pattern can also be etched. Although the patterns are not complete, many small structures have fractures. During the etching process, we observed that, while 3–5 layers of graphene can engrave patterns, they require more than ten repeated etching processes. At the same time, due to the uneven distribution of graphene layers across the entire surface, there may be instances where some parts of the same small circular hole are engraved first, while other parts are not fully engraved, resulting in some rough edges that cannot be engraved.

To address the issue of burrs not being removed during the etching process, we continue to reduce the number of graphene layers from 3–5 layers to a single layer. Compared to the high suspension success rate of few-layer graphene, the suspension success rate for single-layer graphene is relatively low, whether using the back float method or high-temperature annealing method to remove PMMA. Moreover, graphene fails to suspend on large-sized pore sizes. The successfully suspended graphene is mainly concentrated on small pore sizes of 5–10 μm. After multiple parameter adjustments and optimizations, this paper presents the final FIB etching parameters for single-layer suspended graphene, as shown in Table 3.

**Figure 11:**
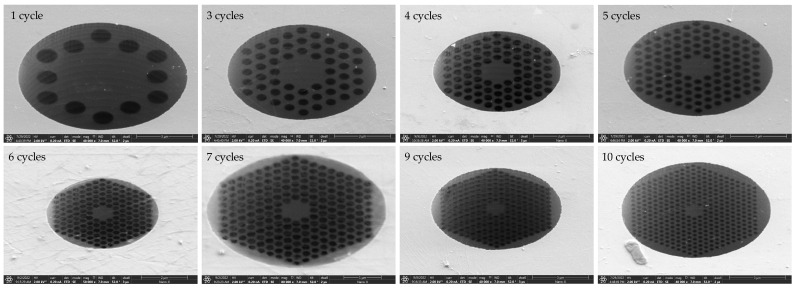
FIB etching results of single-layer graphene obtained by back float method.

The ion beam current used in Table 3 is 10 pA, and the ion beam voltage is 30 kV. These values represent the minimum etching current and voltage that can be set by the FEI Scios Dual Beam instrument for structural etching. Through three repeated scans and etches, phononic crystal soft-supported graphene resonators with varying cycles and pore sizes were obtained. The etching results are depicted in Figure 11. The single-layer suspended graphene used in this etching was obtained by removing PMMA using the back float method.

#### 3.3.3. Suspended Release Mode

**Figure 12:**
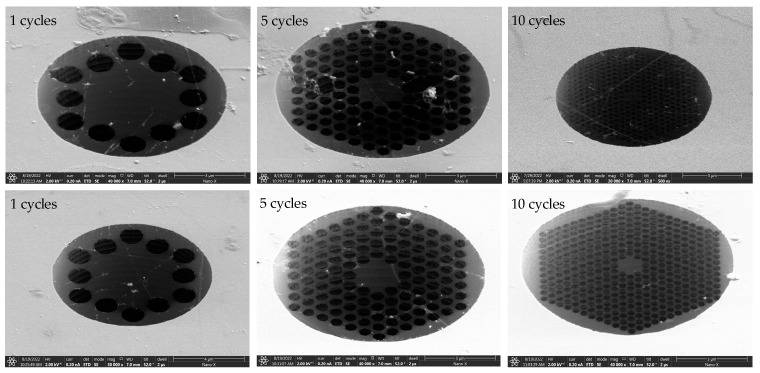
FIB etching results of single-layer graphene obtained by back high-temperature annealing.

The results of FIB etching on the single-layer graphene obtained through high-temperature annealing are depicted in Figure 12. Compared with the etching results obtained using the back float method in Figure 11, the single-layer suspended graphene obtained through high-temperature annealing exhibits a less effective etching and a more contaminated graphene surface. Although the high-temperature annealing method can enhance the success rate of graphene suspension, it also increases the stress on graphene, making it more susceptible to fracturing during FIB etching and, consequently, leading to a lower etching success rate.

#### 3.3.4. Etching Cycles

**Figure 13:**
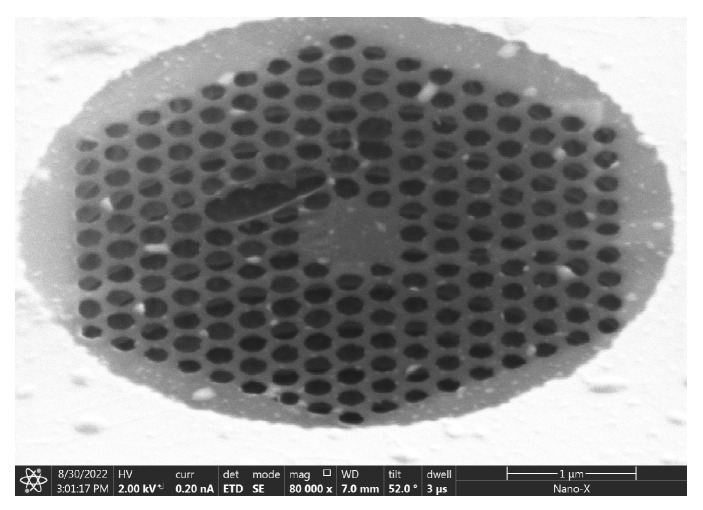
FIB etching results of single-layer graphene for 6 times.

In fact, the number of FIB etching cycles also significantly impacts the etching results. As the number of etching cycles increases, the size of the circular hole near the center also increases, sometimes exceeding the size specified by the etching pattern. As shown in Figure 13, the outcome of six repeated etching cycles reveals that the diameter of the circular hole in the central region is larger than that near the edge. At the same time, as the circular hole near the central region continued to expand, the film was ultimately torn. For single-layer graphene, the optimal etching cycles is three.

## 4. Discussion

### 4.1. Optical Excitation and Detection System Based on Fabry–Pérot Interference Principle

**Figure 14:**
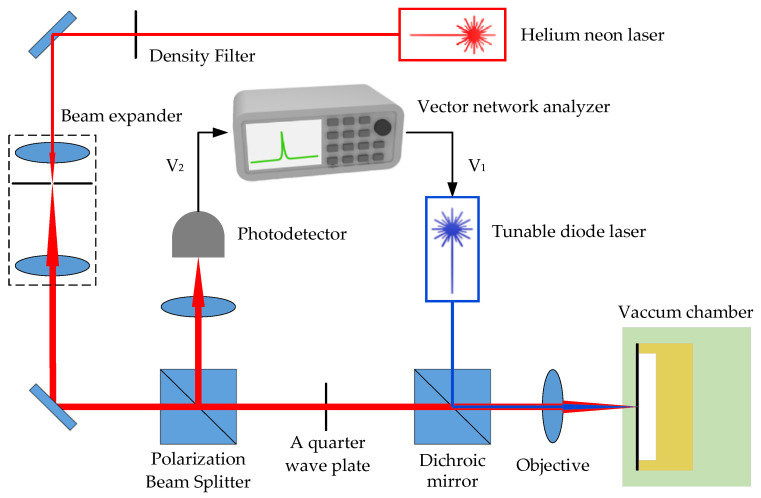
Schematic diagram of an all-optical excitation and detection device based on Fabry–Pérot interference principle.

To verify the effect of improving the quality factor of phononic crystal soft-supported graphene resonators, an optical excitation and detection platform based on the Fabry–Pérot interference principle was designed to test and analyze the resonators’ characteristics. Figure 14 depicts a schematic diagram of an optical excitation and detection system based on the principle of Fabry–Pérot interference. The vibration detection of graphene resonators utilizes a linearly polarized helium–neon laser with a wavelength λ of 633 nm, and the output power is attenuated by a neutral density filter (NDF). The modulated beam is expanded using two 3× lenses to match the aperture of the objective lens. Then, the expanded beam passes through a polarization beam splitter (PBS) and a quarter wave plate (λ/4). Finally, it shot onto the graphene film. After being reflected by the entire resonator, the reflected light passes through the quarter wave plate again and is directed onto the photodetector by the PBS. The presence of red lines and arrows indicates the detection light throughout the process.

To drive the graphene resonator to vibrate at the resonant frequency, a blue diode laser with a radio frequency (RF) modulation intensity function is used as the excitation light source based on photothermal excitation. The excitation light and detection light are coupled through a dichroic mirror (DM), and then directed towards the graphene film simultaneously. The graphene resonator is placed in a small vacuum chamber with a transparent window to minimize the effects of gas damping loss.

The photodetector transmits the detected photoelectric signal to the vector network analyzer (VNA), which modulates the transmission gain of the blue excitation laser. Finally, to precisely focus the blue and red lasers on the graphene resonator, the vacuum chamber is mounted on a three-dimensional control table, allowing for position control in the x, y, and z directions. During the alignment process, CCD cameras and LED lighting are used to monitor the position of the graphene film and laser spot.

### 4.2. Experimental Verification of Energy Dissipation Suppression in Phononic Crystal Soft-Supported Structure

When a laser is irradiated onto the surface of graphene thin films, three effects occur: photon pressure, stress changes caused by the concentration of photogenerated carriers, and photothermal effects (thermal stress caused by light). The primary factor responsible for the vibration of graphene films is the photothermal effect. As mentioned in the previous section, diode lasers are used to excite graphene resonators. The laser’s output power is modulated by radio frequency signals. When light is incident on the resonant cavity, it causes periodic heating and cooling of the graphene film, leading to forced vibration at the driving frequency.

Using finite element simulation with COMSOL, this paper integrates the “Solid Mechanics” module and the “Solid Heat Transfer” module to simulate the thermal coupling in solids and the resulting thermal expansion and contraction behavior. The thermal properties of graphene in finite element simulations are presented in Table 4.

The heat generated by the photothermal effect in the simulation that is absorbed by the graphene film can be expressed as [9]:

(5) $$Q_{n}=\frac{nP\partial }{\pi r_{0}^{2}}\exp\left(-\frac{r^{2}}{r_{0}^{2}}\right)$$

in which *Q_n_* is the heat absorbed by the graphene film, *n* is the number of layers of the graphene film, *P* is the laser output power, ∂=2.3% is the absorbance of the single-layer graphene film, *r* is the radius of the graphene film, and *r*_0_ is the radius of the laser beam. In the actual simulation, a single-layer graphene with a six-cycle phononic crystal structure and a pore size of 2.5 μm is selected. The simulation “heat source” is defined in the “solid heat transfer” module, and the absorbed heat is determined by Equation (5). Finally, choose the “steady-state study” and “characteristic frequency study” modules to enable the photothermal driving of graphene thin films.

**Figure 15:**
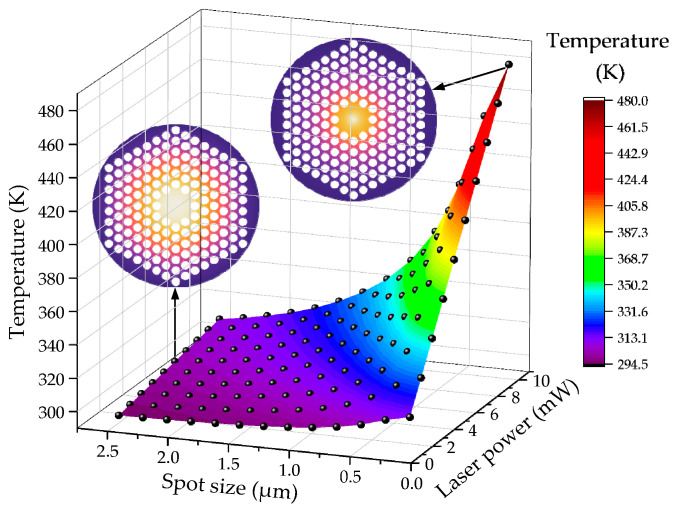
Diagram of the relationship between the center point temperature of graphene resonator and laser power and spot size.

According to Equation (5), the total energy absorbed by the graphene film is closely related to the laser output power *P* and spot radius *r*_0_. It should be noted that, if the center temperature of the film is too high, the suspended graphene film may partially burn out, resulting in resonator failure. Generally, the presence of prestress during the transfer of graphene films affects the damage threshold of the films, which is related to the preparation and transfer process. To prevent physical damage to the graphene films mentioned above, the output power is kept below 10 mW during the simulation process. Therefore, in this paper, *P* and *r*_0_ are set within the range of 1–10 mW and 0.1–2.5 μm, respectively. Figure 15 illustrates the correlation between the center point temperature of graphene thin films, laser power, and spot size. The laser power is measured in 1 mW intervals within the range of 1–10 mW, while the spot size (radius) is measured in 0.2 μm intervals within the range of 0.1–2.5 μm. The black sphere in the figure represents the value point and is interpolated to create a three-dimensional colored surface. As the radius of the laser spot decreases and the laser power increases, the temperature at the center of the graphene film increases. The two surface temperature distribution illustrations in the figure correspond to the cases of 0.1 μm and 10 mW, 2.5 μm, and 6 mW, respectively. It can be observed that, when the laser spot is relatively small, the temperature will primarily concentrate at the center of the film. When the size of the laser spot is comparable to the size of the graphene film, the temperature will be distributed more evenly across the entire plane. The finite simulation provides guidance for selecting the appropriate laser power and spot size for future real experiments. The laser power should not be too high, and the spot size should not be too small to prevent an excessively high central temperature.

**Figure 16:**
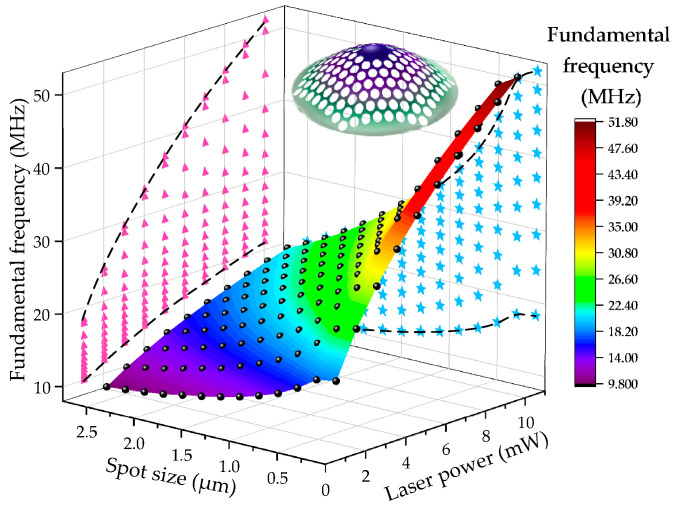
Diagram of the relationship between the fundamental frequency of graphene resonator and laser power and spot size. Pink triangles represent the *f*-*P* projection, while blue pentagons indicate the *f*-*r*_0_ projection.

In fact, variations in laser parameters (output power and spot radius) have a significant influence on the resonant frequency *f* driven by photothermal effects. When using the same laser power and spot size interval and range, the simulated response of first-order modal frequency (fundamental frequency) is shown in Figure 16. As the spot radius decreases and the laser power increases, the frequency of the first-order mode of the graphene resonator increases. The illustration depicts the first-order mode of the phononic crystal soft-supported graphene resonator. Next, we project the *f*-*P* and *f*-*r*_0_ curves from Figure 16 onto the corresponding planes, which are represented by pink triangles and blue pentagons, respectively. By fitting the curves, it can be observed from the *f*-*P* projection that the fundamental frequency exhibits weak nonlinearity with the change in laser output power. In the *f*-*r*_0_ projection, the *f*-*r*_0_ curve exhibits greater nonlinearity compared to the *f*-*P* curve. However, as the laser output power decreases, the nonlinearity gradually diminishes.

## 5. Conclusions

In response to the current problems of the low quality factor in graphene resonators at room temperature, this article investigates the energy dissipation distribution characteristics of graphene resonators based on the established energy dissipation dilution theoretical model. The results indicate that energy dissipation is primarily concentrated at the fixed support edge. To address this issue, a new mechanism for suppressing phonon tunneling losses in graphene resonators using phononic crystal soft-supported structures is proposed under the guidance of the energy dissipation dilution theory. Additionally, the graphene resonator with a phononic crystal soft-supported structure is designed and fabricated. In the fabrication process, the study addresses the low success rate of graphene suspension release by employing the back float method and high-temperature annealing method to improve the success rate. After successfully suspending graphene, during the FIB etching process, the suspended graphene is more susceptible to fracturing and deformation. Therefore, our primary focus is on investigating the impact of surface wrinkles, the number of graphene layers, the mode of suspended release, and the etching cycles to optimize etching parameters for improving the etching success rate.

Finally, we have fabricated phononic crystal soft-supported graphene resonators with varying cycles and pore sizes using single-layer graphene. At last, a platform for optical excitation and detection based on the Fabry–Pérot interference principle has been designed. Based on the platform, we used finite element simulation to investigate the impact of laser power and spot size on the central temperature and fundamental frequency. The results showed that, as the spot radius decreases and the laser power increases, the central temperature and fundamental frequency of phononic crystal soft-supported graphene resonator increase.

Compared to previous research in the field of energy dissipation in graphene resonators, which includes ohmic loss, thermoelastic losses, nonlinear loss, friction loss, and so on, we attribute the energy dissipation to phonon tunneling loss, also known as anchoring or fixed loss. Previous studies have achieved phononic crystal structures on silicon nitride material. In this article, the phonoinic crystal soft-supported structure has been realized on much thinner graphene material. Due to current experimental limitations, the FIB equipment used in this article is based on the Ga ion. However, when etching thin graphene, it is more appropriate to use smaller H ion, a topic that will be explored in future research. The critical point drying method will also be tested to achieve a higher yield when removing PMMA to suspend graphene. In a future work, we will select specific optical equipment based on functional requirements to build a physical platform according to the designed optical system, and debug the optical path to achieve excitation and detection functions. Furthermore, detailed experimental verification will be conducted to determine whether a phononic crystal soft-supported structure can effectively enhance the quality factor of graphene resonators by suppressing energy dissipation.

## Figures and Tables

**Table 1:** Material parameter.

Material	Graphene	Silicon Nitride
Density *ρ* (kg/m^3^)	2250	3200
Thickness *h* (nm)	0.335	30
Young’s modulus *E*_1_ (GPa)	1000	270
Poisson’s ratio *ν*	0.165	0.27

**Table 2:** Success rates of graphene suspension on different pore sizes through high-temperature annealing.

Pore Size (μm)	Suspension Success Rate
300	0
200	8.3%
150	16.7%
100	40.7%
80	68.8%
60	72%
50	89.8%
40	91.2%
30	93.2%
5–20	95–99%

**Table 3:** FIB etching parameters for single-layer suspended graphene.

Parameter	Value
Ion beam voltage	30 kV
Ion beam current	10 pA
Magnification factor	20,000
Etching depth	0.39 pm
Etching duration	2 s
Etching cycles	3

**Table 4:** Graphene thermal parameters.

Parameter	Value
Thermal conductivity	5000 W/(m·K)
Specific heat capacity	700 J/(kg·K)
Coefficient of thermal expansion	−8 × 10^−6^ 1/K

## Data Availability

The data presented in this study are available upon request from the corresponding author.
